# Extent of surgical repair and outcomes after surgery for type A aortic dissection

**DOI:** 10.1093/bjsopen/zraf003

**Published:** 2025-03-12

**Authors:** Fausto Biancari, Daniele Fileccia, Luisa Ferrante, Timo Mäkikallio, Tatu Juvonen, Mikko Jormalainen, Giovanni Mariscalco, Zein El-Dean, Matteo Pettinari, Javier Rodriguez Lega, Angel G Pinto, Andrea Perrotti, Francesco Onorati, Konrad Wisniewski, Till Demal, Petr Kacer, Jan Rocek, Dario Di Perna, Igor Vendramin, Daniela Piani, Mauro Rinaldi, Eduard Quintana, Robert Pruna-Guillen, Sven Peterss, Joscha Buech, Caroline Radner, Manoj Kuduvalli, Amer Harky, Antonio Fiore, Michele D’Alonzo, Angelo M Dell’Aquila, Giuseppe Gatti, Lenard Conradi, Andrea Ballotta, Mark Field

**Affiliations:** Department of Cardiovascular Surgery, Centro Cardiologico Monzino IRCCS, Milan, Italy; Department of Cardiovascular Surgery, Centro Cardiologico Monzino IRCCS, Milan, Italy; Cardiac Surgery, Molinette Hospital, University of Turin, Turin, Italy; Department of Medicine, South-Karelia Central Hospital, University of Helsinki, Lappeenranta, Finland; Heart and Lung Center, Helsinki University Hospital, University of Helsinki, Helsinki, and Faculty of Medicine, University of Oulu, Oulu, Finland; Heart and Lung Center, Helsinki University Hospital, University of Helsinki, Helsinki, and Faculty of Medicine, University of Oulu, Oulu, Finland; Department of Cardiac Surgery, Glenfield Hospital, Leicester, UK; Department of Cardiac Surgery, Glenfield Hospital, Leicester, UK; Department of Cardiac Surgery, Ziekenhuis Oost Limburg, Genk, Belgium; Cardiovascular Surgery Department, University Hospital Gregorio Marañón, Madrid, Spain; Cardiovascular Surgery Department, University Hospital Gregorio Marañón, Madrid, Spain; Department of Thoracic and Cardiovascular Surgery, University of Franche-Comte, Besancon, France; Division of Cardiac Surgery, University of Verona Medical School, Verona, Italy; Department of Cardiothoracic Surgery, University Hospital Muenster, Muenster, Germany; Department of Cardiovascular Surgery, University Heart and Vascular Center Hamburg, Hamburg, Germany; Department of Cardiac Surgery, Charles University and University Hospital Kralovske Vinohrady, Prague, Czech Republic; Department of Cardiac Surgery, Charles University and University Hospital Kralovske Vinohrady, Prague, Czech Republic; Department of Cardiac Surgery, Centre Hospitalier Annecy Genevois, Epagny Metz-Tessy, France; Cardiothoracic Department, Azienda Sanitaria Universitaria Friuli Centrale, Udine, Italy; Cardiothoracic Department, Azienda Sanitaria Universitaria Friuli Centrale, Udine, Italy; Cardiac Surgery, Molinette Hospital, University of Turin, Turin, Italy; Department of Cardiovascular Surgery, Hospital Clínic de Barcelona, University of Barcelona, Barcelona, Spain; Department of Cardiovascular Surgery, Hospital Clínic de Barcelona, University of Barcelona, Barcelona, Spain; Department of Cardiac Surgery, LMU University Hospital, Ludwig Maximilian University, Munich, Germany; Department of Cardiac Surgery, LMU University Hospital, Ludwig Maximilian University, Munich, Germany; German Centre for Cardiovascular Research, Partner Site Munich Heart Alliance, Munich, Germany; Department of Cardiac Surgery, LMU University Hospital, Ludwig Maximilian University, Munich, Germany; German Centre for Cardiovascular Research, Partner Site Munich Heart Alliance, Munich, Germany; Liverpool Centre for Cardiovascular Sciences, Liverpool Heart and Chest Hospital, Liverpool, UK; Liverpool Centre for Cardiovascular Sciences, Liverpool Heart and Chest Hospital, Liverpool, UK; Department of Cardiac Surgery, Hôpitaux Universitaires Henri Mondor, Assistance Publique-Hôpitaux de Paris, Créteil, France; Mondor Biomedical Research Institute, Université Paris Est Créteil, Inserm, CEpiA Team, Créteil, France; Department of Cardiac Surgery, Hôpitaux Universitaires Henri Mondor, Assistance Publique-Hôpitaux de Paris, Créteil, France; Department of Cardiothoracic Surgery, University Hospital Muenster, Muenster, Germany; Department of Cardiac Surgery, Martin Luther University Halle-Wittenberg, Halle, Germany; Division of Cardiac Surgery, Cardio-thoracic and Vascular Department, Azienda Sanitaria Universitaria Giuliano Isontina, Trieste, Italy; Department of Cardiac Surgery, Cologne University Hospital, Cologne, Germany; Department of Cardiovascular Surgery, Centro Cardiologico Monzino IRCCS, Milan, Italy; Liverpool Centre for Cardiovascular Sciences, Liverpool Heart and Chest Hospital, Liverpool, UK

## Abstract

**Background:**

Acute Stanford type A aortic dissection is a severe emergency condition that, if left untreated, is associated with a high mortality rate. The extent of surgical repair may impact the outcomes of these patients.

**Method:**

Patients operated for acute type A aortic dissection from a multicentre European registry were included. Patients were categorized based on the following types of surgical intervention: isolated ascending aortic replacement, ascending aortic replacement with concomitant aortic valve replacement, aortic root replacement, partial or total arch replacement, and partial or total arch replacement with concomitant aortic root replacement. The primary outcome was mortality rate, both in-hospital and at 10 years. Secondary outcomes were acute kidney injury requiring dialysis, neurological complications, a composite endpoint including in-hospital death, neurological complications and/or dialysis, and proximal endovascular or surgical aortic re-operations at 10 years.

**Results:**

3702 patients were included. The adjusted risk of in-hospital mortality was higher in all subsets of patients compared to those who underwent isolated ascending aortic replacement. The adjusted rates of in-hospital mortality ranged from 16.4% (95% c.i. 15.3 to 17.4) among patients who underwent isolated ascending aortic replacement to 27.7% (95% c.i. 23.3 to 31.2) among those who underwent aortic arch and concomitant aortic root replacement. The adjusted risks of neurological complications, renal replacement therapy and of the composite endpoint were significantly higher in patients who underwent partial/total aortic arch replacement. The adjusted risk estimates of 10-year mortality rate were markedly higher in patients who underwent partial/total aortic arch replacement with or without concomitant aortic root replacement. Extensive aortic repair did not significantly reduce the risk of distal or proximal aortic reoperations.

**Conclusion:**

These findings suggest that, when feasible, limiting the extent of aortic replacement for acute type A aortic dissection may be beneficial in reducing mortality rate and major complications both in the short and long term.

**Trial Registration:**

ClinicalTrials.gov identifier: NCT04831073.

## Teaser

When feasible, limiting the extent of aortic replacement for acute type A aortic dissection may be beneficial in reducing mortality and major complications both in the short and long term.

## Introduction

Acute Stanford type A aortic dissection (TAAD) is a severe emergency that, if left untreated, is associated with a high mortality rate^1^. Surgery is the only treatment for risk reduction of mortality of TAAD^2,3^. The pillars of surgery for acute TAAD are the excision of the primary entry tear, repair of aortic valve insufficiency, and restoration of true lumen blood flow to the downstream aorta^4^. The extent of surgery for acute TAAD is a matter of debate because limited resection of the dissected aorta may potentially result in late aortic dissection-related complications^5^. However, there is also evidence that primary tear resection alone does not impact the midterm outcome after surgery for acute TAAD^6^. Notably, the more extensive the surgical resection at primary surgery, the higher the risk of early adverse events in these patients. As data on the prognostic impact of extensive surgery for TAAD are scarce^7^, this issue was investigated in the present multicentre study.

## Methods

The European Registry of TAAD (ERTAAD) is a registry that included comprehensive information on patients who have undergone aortic surgery for acute TAAD at 18 cardiac surgery centres in seven European countries (Belgium, Czech Republic, Finland, France, Germany, Italy, and the UK) between January 2005 and March 2021. The Ethical Review Board of the Helsinki University Central Hospital, Finland (April 21, 2021, diary no. HUS/237/2021) and that of each participating hospital approved this study. The ERTAAD was registered in ClinicalTrials.gov with the identifier NCT04831073 (https://clinicaltrials.gov/study/NCT04831073).

The inclusion criteria of the ERTAAD registry were patients with acute TAAD; patients > 18 years old; onset of symptoms within 7 days prior to surgery; primary surgical repair of acute TAAD; any other major cardiac surgical procedure concomitant with surgery for TAAD. The exclusion criteria were retrograde TAAD; concomitant endocarditis; TAAD secondary to blunt or penetrating chest trauma^8^.

This study followed the STROBE guidelines^9^.

Details on the definition criteria of clinical, operative and outcome variables have been previously reported^8^. The urgency of the procedure was defined as follows: urgent procedure—scheduled procedure performed in paucisymptomatic patients with stable haemodynamic conditions during the index hospitalization from the next working day from admission; emergency procedure grade 1—procedure performed in patients with stable haemodynamic conditions, even if with the use of inotropes, and without clinically evident malperfusion before the beginning of the next working day; emergency procedure grade 2—procedure performed in patients with haemodynamic instability despite the use of inotropes and/or any clinically evident malperfusion before the beginning of the next working day (no cardiopulmonary resuscitation with chest compression required); salvage procedure grade 1—procedure performed in patients requiring cardiopulmonary resuscitation with external chest compressions and/or open chest cardiac massage between induction of anaesthesia and initiation of cardiopulmonary bypass; salvage procedure grade 2—procedure performed in patients requiring cardiopulmonary resuscitation with external chest compressions en route to the operating theatre or before induction of anaesthesia.

The study population was categorized according to the following types of surgical intervention: isolated ascending aortic replacement, ascending aortic replacement with concomitant aortic valve replacement, aortic root replacement, partial or total arch replacement, and partial or total arch replacement with concomitant aortic root replacement. Isolated ascending aortic replacement included patients who underwent only repair of the ascending aorta with or without resection of the inner curve of the aortic arch, that is the hemiarch repair. Partial aortic arch referred to aortic arch resection with concomitant reimplantation of at least one epiaortic vessel. Aortic root replacement referred to the Bentall procedure, the David procedure, the Yacoub procedure, and the Florida sleeve procedure. Patients who underwent partial or total aortic arch replacement with concomitant aortic valve replacement were excluded from the present analysis because of the limited number of this subset of patients.

The primary outcomes of this study were in-hospital mortality rate, that is all-cause mortality rate during the index hospitalization, and 10-year all-cause mortality rate. The secondary outcomes of this analysis were acute kidney injury requiring dialysis and neurological complications (ischaemic or haemorrhagic stroke and/or global brain ischaemia) occurring during the index hospitalization as well as a composite endpoint including in-hospital death, neurological complications, and/or dialysis. Distal and proximal endovascular or surgical aortic re-operations at 10 years were the other secondary outcomes.

## Statistical analysis

Categorical variables are reported as counts and percentages, whereas continuous variables are reported as means and standard deviations or median and interquartile range. The chi-square test and Kruskal–Wallis test were used to evaluate differences between the study groups. Kaplan–Meier’s method with the log-rank test and the competing risk analysis considering mortality as the competing event were used to evaluate late mortality and late aortic re-operation, respectively. Competing risk analysis was performed with the Fine–Gray test, considering mortality as the competing event. Multivariable analysis was performed using the multilevel mixed-effects logistic regression and parametric survival regression, considering the cluster effect of the participating hospital. Multivariable analyses considered the following covariates: age, sex, estimated glomerular filtration rate according to the CKD-EPI equation, genetic syndromes, family history of aortic dissection and/or aneurysm, prior cardiac surgery, iatrogenic TAAD, diabetes, prior stroke, pulmonary disease, extracardiac arteriopathy, preoperative cardiac massage, invasive mechanical ventilation, urgency of the procedure, cerebral malperfusion, spinal malperfusion, renal malperfusion, mesenteric malperfusion, peripheral malperfusion, tear in the aortic arch, DeBakey type 1 aortic dissection, bicuspid aortic valve and concomitant coronary surgery. Adjusted risk estimates are reported as ORs, HRs, and sub-distributional HR (SHR) with their 95% confidence intervals. Multivariable adjusted rates of in-hospital mortality with their 95% c.i. were calculated by dividing the observed number of patients who died during the index hospitalization by the expected number of patients who died during the index hospitalization and by multiplying this ratio by the mean in-hospital mortality rate of the entire series. The expected numbers of in-hospital deaths were estimated using multilevel mixed-effects logistic regression. Statistical significance was set at *P* < 0.05. Statistical analyses were performed using the Stata (version 15.1, StataCorp LLC, College Station, Texas, USA) statistical software.

## Results

Of 3735 patients included in the ERTAAD, 3702 patients fulfilled the inclusion and exclusion criteria of this study. The characteristics and preoperative data of patients of the study groups are summarized in *Table 1*.

**Table 1:** Preoperative characteristics of each type of surgery group

Baseline characteristics	Isolated supracoronary aortic replacement (*N* = 1963)	Ascending aortic replacement and AVR (*N* = 160)	Aortic root replacement (*N* = 863)	Partial/total aortic arch replacement (*N* = 507)	Partial/total aortic arch replacement and aortic root replacement (*N* = 209)	*P*
Age (years), mean(s.d.)	66.3(11.8)	68.3(12.2)	58.3(13.6)	61.0(13)	56.0(12)	<0.001
Male sex	1241 (63.2)	112 (70.0)	680 (78.8)	371 (73.2)	171 (81.8)	<0.001
eGFR, ml/min/1.74 m^2^, mean(s.d.)	67(22)	63(22)	74(24)	70(24)	74(25)	<0.001
Genetic syndromes	11 (0.6)	1 (0.6)	42 (4.9)	7 (1.4)	15 (7.2)	<0.001
Family history of aortic dissection/aneurysm	89 (4.5)	7 (4.4)	77 (8.9)	32 (6.3)	19 (9.1)	<0.001
Prior cardiac surgery	71 (3.6)	5 (3.1)	24 (2.8)	13 (2.6)	3 (1.4)	0.356
Iatrogenic TAAD	79 (4.0)	6 (3.8)	12 (1.4)	3 (0.6)	1 (0.5)	<0.001
Diabetes	104 (5.3)	6 (3.8)	45 (5.2)	21 (4.2)	4 (1.9)	0.204
Prior stroke	81 (4.1)	10 (6.3)	32 (3.7)	17 (3.4)	4 (1.9)	0.255
Pulmonary disease	193 (9.8)	8 (5.0)	74 (8.6)	31 (6.1)	10 (4.8)	0.006
Extracardiac arteriopathy	118 (6.0)	12 (7.5)	30 (3.5)	33 (6.5)	5 (2.4)	0.007
Preoperative cardiac massage	91 (4.6)	6 (3.8)	44 (5.1)	21 (4.1)	6 (2.9)	0.658
Invasive mechanical ventilation	194 (9.9)	13 (8.1)	70 (8.1)	49 (9.7)	17 (8.1)	0.571
**Urgency of the procedure**						<0.001
Urgent	270 (13.8)	26 (16.3)	106 (12.3)	77 (15.2)	30 (14.4)	
Emergency 1	837 (42.6)	76 (47.5)	429 (49.7)	177 (34.9)	94 (45.0)	
Emergency 2	767 (39.1)	52 (32.5)	285 (33.0)	234 (46.2)	79 (37.8)	
Salvage 1	45 (2.3)	3 (1.9)	28 (3.2)	15 (3.0)	5 (2.4)	
Salvage 2	44 (2.2)	3 (1.9)	15 (1.7)	4 (0.8)	1 (0.5)	
Cerebral malperfusion	449 (22.9)	30 (18.8)	156 (18.1)	126 (24.9)	51 (24.4)	0.013
Spinal malperfusion	40 (2.0)	0 (0)	15 (1.7)	17 (3.4)	3 (1.4)	0.074
Renal malperfusion	197 (10.0)	12 (7.5)	66 (7.6)	72 (14.2)	11 (5.3)	<0.001
Mesenteric malperfusion	95 (4.8)	2 (1.3)	29 (3.4)	26 (5.1)	7 (3.3)	0.086
Peripheral malperfusion	278 (14.2)	17 (10.6)	116 (13.4)	96 (18.9)	34 (16.3)	0.023
Tear in the aortic root at surgery	198 (10.1)	31 (19.4)	290 (33.6)	51 (10.1)	49 (23.4)	<0.001
Tear in the aortic arch at surgery	249 (12.7)	16 (10.0)	47 (5.4)	220 (43.4)	67 (32.1)	<0.001
DeBakey type I aortic dissection	1639 (83.5)	114 (71.3)	660 (76.5)	489 (96.4)	204 (97.4)	<0.001
Bicuspid aortic valve	26 (1.3)	15 (9.4)	84 (9.7)	2 (0.4)	17 (8.1)	<0.001
CABG	124 (6.3)	10 (6.3)	141 (16.3)	32 (6.3)	30 (14.4)	<0.001
Myocardial ischaemic time (min), mean(s.d.)	93(39)	128(52)	157(55)	136(54)	208(66)	<0.001
Cardiopulmonary bypass time (min), mean(s.d.)	186(68)	225(74)	255(94)	251(80)	321(99)	<0.001

Values are *n* (%) unless otherwise indicated. CABG, coronary artery bypass grafting; eGFR, estimated glomerular filtration rate according to the CKD-EPI equation; TAAD, type A aortic dissection; AVR, aortic valve replacement.

The decision to keep patients who underwent the frozen elephant trunk procedure within the group of patients who underwent partial or total aortic arch replacement was based on the results of preliminary statistical analyses, which showed that the frozen elephant trunk procedure did not have a higher risk of in-hospital mortality (adjusted OR 1.029, 95% c.i. 0.625 to1.695) or 10-year mortality (adjusted HR 1.206, 95% c.i. 0.857 to 1.699).

Aortic root and aortic arch replacement procedures were performed more frequently in younger patients with preserved renal function and genetic syndromes. On the contrary, patients who underwent isolated ascending aortic replacement were significantly older, had lower estimated glomerular filtration rates, and a higher prevalence of pulmonary disease and extracardiac arteriopathy.

Tear in the aortic arch at surgery was present in 220 (43.4%) patients with partial or total arch replacement and in 67 (32.1%) patients with partial or total arch replacement and concomitant aortic root replacement (*Table 1*). Myocardial ischaemia time and duration of cardiopulmonary bypass increased significantly along with the extent of surgery (*Table 1*).

**Fig. 1:**
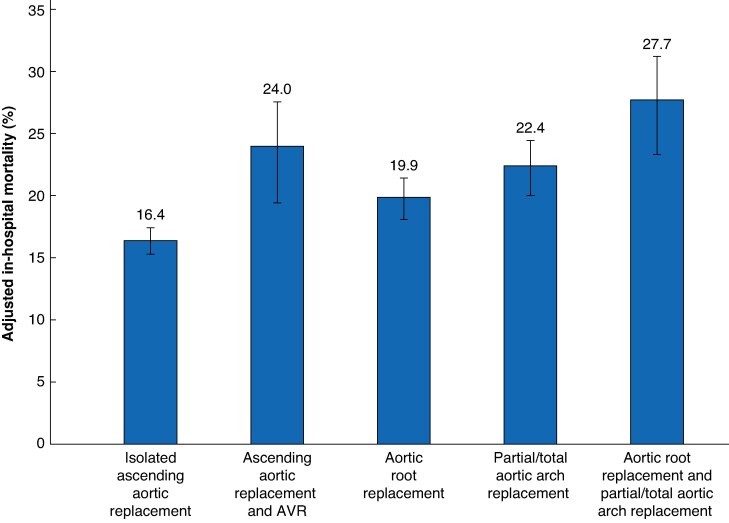
Multivariable adjusted in-hospital mortality between the study groups with their 95% confidence intervals (marked with lines)

In-hospital mortality rate was significantly lower in patients who underwent isolated ascending aortic replacement (*Table 2*). The risk of in-hospital mortality was significantly higher in all the other subsets of patients who underwent more extensive surgery. The adjusted rates of in-hospital mortality ranged from 16.4% (95% c.i. 15.3 to 17.4) among patients who underwent isolated ascending aortic replacement to 27.7% (95% c.i. 23.3 to 31.2) among those who underwent aortic arch and concomitant aortic root replacement (*Fig. 1*). The adjusted risks of neurological complications, renal replacement therapy and composite endpoint were significantly higher in patients who underwent aortic arch replacement with or without concomitant aortic root replacement (*Table 2*).

**Table 2:** Early and late outcomes according to different types of aortic repair for type A aortic dissection

Outcomes	Isolated supracoronary aortic replacement	Ascending aortic replacement and AVR	Aortic root replacement	Partial/total aortic arch replacement	Partial/total aortic arch replacement and aortic root replacement
**In-hospital outcomes**					
Mortality	311 (15.8)	36 (22.5)	149 (17.3)	101 (19.9)	45 (21.5)
Adjusted risk (OR (95% c.i.))	Reference	1.89 (1.21,2.96)	1.56 (1.19,2.04)	1.79 (1.32,2.44)	2.60 (1.70,3.99)
Neurological complications	355 (18.1)	27 (16.9)	137 (15.9)	111 (21.9)	46 (22.0)
Adjusted risk (OR (95% c.i.))	Reference	0.95 (0.59,1.55)	0.88 (0.68,1.14)	1.34 (1.01,1.80)	1.59 (1.06,2.39)
Renal replacement therapy	296 (15.1)	19 (11.9)	94 (10.9)	105 (20.7)	37 (17.7)
Adjusted risk (OR (95% c.i.))	Reference	0.73 (0.40,1.30)	0.78 (0.58,1.06)	1.55 (1.47,2.10)	1.49 (0.95,2.34)
Composite endpoint	683 (34.8)	60 (37.5)	285 (33.1)	230 (45.4)	91 (43.5)
Adjusted risk (OR (95% c.i.))	Reference	1.15 (0.78,1.70)	1.11 (0.89,1.37)	1.91 (1.50,2.44)	2.09 (1.47,2.97)
**10-year outcomes**					
Mortality	660 (49.1)	61 (51.9)	273 (42.6)	193 (54.3)	72 (45.8)
Adjusted risk (HR (95% c.i.))	Reference	1.14 (0.85,1.54)	1.25 (1.06,1.47)	2.02 (1.68,2.43)	2.03 (1.53,2.70)
Repeat proximal aortic operation	57 (4.6)	5 (6.1)	26 (4.6)	11 (3.1)	7 (4.8)
Adjusted risk (SHR (95% c.i.))	Reference	1.16 (0.47,2.90)	0.62 (0.36,1.05)	0.73 (0.38,1.39)	0.95 (0.42,2.16)
Repeat distal aortic operation	90 (7.3)	7 (7.9)	48 (8.0)	33 (10.0)	11 (8.1)
Adjusted risk (SHR (95% c.i.))	Reference	1.08 (0.50,2.31)	1.00 (0.68,1.43)	1.15 (0.77,1.72)	0.85 (0.45,1.59)

Outcomes are reported as crude number of events with their frequency, survival rates or cumulative incidences in parentheses. Adjusted risk estimates are OR, HR, and subdistributional hazard ratios (SHR) with their 95% confidence intervals. Composite endpoint includes in-hospital death, neurological complications and/or dialysis. AVR, aortic valve replacement.

**Fig. 2:**
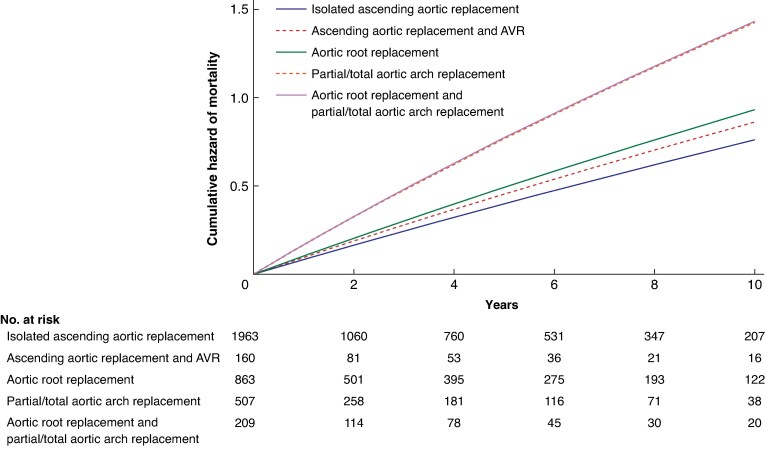
Multivariable adjusted cumulative hazards of mortality in the study groups

The median follow-up was 2.5 years (mean 3.9 years, i.q.r. 6.2, 13 347 persons/year). The number of deaths at 10 years was 1259 (incidence rate 9.4%, 95% c.i. 8.9% to 10.0%). The 10-year crude rate of mortality was 48.1%. The adjusted risk estimates of 10-year mortality were significantly higher in the subsets of patients who underwent aortic root replacement and partial/total aortic arch replacement (*Table 2*, *Fig. 2*). Extensive aortic repair did not significantly reduce the risk either of distal or proximal aortic reoperations (*Table 2*).

## Discussion

The main findings of this multicentre study can be summarized as follows: the more extensive the repair of TAAD, the higher the adjusted rate of in-hospital mortality; the adjusted risk of neurological complications, severe acute kidney injury requiring renal replacement therapy and composite endpoint were significantly higher in patients who underwent partial/total aortic arch repair; aortic arch repair and aortic root repair were associated with significantly increased adjusted risk of 10-year mortality; extensive surgery did not reduce the rates of proximal and distal aortic reoperation; and 60% of patients who underwent partial/total aortic arch replacement did not have an intimal tear localized in the aortic arch.

The outcomes observed in this analysis seem to be the genuine consequence of surgical procedures of increasing extent and complexity. Indeed, the present findings indicated that either aortic root replacement and partial/total aortic arch replacement can be performed with aortic cross clamping time and duration of cardiopulmonary bypass, which are close to or well beyond safe time limits (150 min for aortic cross clamping and 240 min for cardiopulmonary bypass)^10^ (*Table 1*). Duration of myocardial ischaemia (mean 208 ± 66 min) and cardiopulmonary bypass (mean 321 ± 99 min) were excessively long when aortic arch replacement was performed in conjunction with aortic root replacement and resulted in an adjusted rate of in-hospital mortality of 27.7% (95% c.i. 23.3 to 31.2). The shorter duration of myocardial ischaemia and cardiopulmonary bypass time necessary to accomplish an aortic root/aortic valve procedure might explain the lower risk of long-term mortality compared to patients who underwent aortic arch surgery through a reduced risk of early adverse events such as neurological complications and severe acute kidney injury. These findings should be viewed in the light of the relatively young age and low prevalence of co-morbidities in the subset of patients who underwent aortic arch replacement. Notably, 60% of patients underwent aortic arch replacement without the presence of an intimal tear localized in the aortic arch. This finding reflects the current policy of institutions/individual surgeons of resecting the aortic arch based on the extent of the dissection flap in the absence of any entry tear located in the aortic arch^11,12^. This may also apply to the treatment of dissected aortic root. In particular, the frozen elephant trunk procedure is expected to favour the remodelling of the dissected aorta by excluding the entry tears in either the aortic arch or descending aorta, restoring antegrade blood flow in the true lumen and inducing false lumen thrombosis^13^. However, the long-term benefits of total aortic arch replacement in reducing the risk of distal aortic reoperations were not observed in this series, or in a previous multicentre study^14^. Indeed, the present study demonstrated that, when adjusted for baseline characteristics and the cluster effect of the participating hospital was considered, partial/total aortic arch replacement was associated with increased risk of 10-year mortality. Although the data on the cause of late mortality of these patients is not available, it is hypothesized that severe postoperative complications after aortic arch repair in the emergency setting might have a role in their significantly higher rate of 10-year mortality.

The results of this study should be viewed considering a few methodological limitations. First, this is a registry and its nature along with the lack of data on further baseline confounders are the main limitations of this analysis. Second, the study groups differed markedly in several baseline characteristics, which most likely were considered in the pre- and intraoperative decision-making process. Third, the multicentre nature of this study including patients with different referral pathways, surgical strategies and perioperative care might have introduced bias related to interinstitutional differences in the management and outcomes of acute TAAD. To overcome these potential biases, a multilevel mixed-effects regression methodology that considered the cluster effect on the outcomes was used. Fourth, data on aortic-related mortality rate are not available because of the currently reduced frequency of diagnosis at autopsy in most European countries. Finally, the present data demonstrated that the extent of surgical repair might have been dictated by the site of intimal tear and injury of the dissected aortic wall in several, but not all, patients. Therefore, the conclusions of this analysis may only be relevant to those patients in whom limited aortic repair could have been feasible.

The present results suggest that extensive surgical procedures for acute TAAD, particularly when involving the aortic arch, are associated with significantly higher in-hospital and long-term mortality compared to more limited interventions. The risk of aortic reoperations did not differ significantly between patients who underwent extensive surgery and those who had only the ascending aorta replaced.

## Data Availability

The data of this registry are not publicly available due to privacy issues.
